# Non-neutralizing Antibody Responses against VP1 in *Enterovirus A, B, C* and *Rhinovirus A species* among Infants and Children in Shanghai

**DOI:** 10.1038/s41598-018-23683-x

**Published:** 2018-04-03

**Authors:** Yingying Ding, Bing Rui, Caixia Gao, Menghua Xu, Lili Wang, Chunyan Zhao, Jie Bai, Jinhong Wang, Jin Xu, Wei Pan

**Affiliations:** 1 Department of Medical Microbiology and Parasitology, School of Basic Medicine, Second Military Medical University, No. 8 Panshan Road, Shanghai, 200433 China; 2 Department of Clinical Laboratory, Children’s Hospital of Fudan University, 399 Wanyuan Road, Shanghai, 201102 China

## Abstract

The overall non-neutralizing antibody responses against EV infections among infants and children remain unknown. The non-neutralizing antibody responses against VP1 of *EV-A* species (Enterovirus 71 (EV71), Coxsackievirus A16 (CA16)), *EV-B* species (Coxsackievirus B3 (CB3)), *EV-C* species (Poliovirus 1 (PV1)) and *RV-A* species (Rhinovirus A N13 (RV13)) were detected and analyzed using a novel evolved immunoglobulin-binding molecule (NEIBM)-based ELISA among infants and children aged 1 day to 6 years in Shanghai. The anti-VP1 reactivity against these EVs changed similarly in an age-related dynamic: being high level in the 1–28-day age group, declining to the lowest level in the 1–12-month age group, gradually increasing to the peak level in the 13–60-month age group, and remarkably declining in the 61–72-month age group, which reflects the conversion from maternally-derived to primary antibody responses. The anti-RV13 VP1 antibodies were demonstrated at the highest level, with anti-CB3 and PV1 VP1 antibodies at the second highest level and anti-CA16 and EV71 VP1 antibodies at the lowest level. These findings are the first to describe the overall non-neutralizing antibody responses against VP1 of the *EV-A, B, C* and *RV-A* viruses among the infants and children and could be helpful for further understanding the ubiquitous EV infections among children.

## Introduction

A large number of enteroviruses (EVs), belonging to a genus of the *Picornaviridae* family, are among the most common viruses infecting humans. These EVs are phylogenically divided into 13 species: enterovirus A, B, C, D, E, F, G, H, I and J (*EV A-J*), and rhinovirus A, B and C (*RV A-C*). Seven of these species (*EV-A*, *EV-B*, *EV-C*, *EV-D*, *RV-A*, *RV-B*, *RV-C*) infect humans and cause a wide spectrum of illnesses. *EV-A* consists of 25 types, including CA16, EV71 and simian (or baboon) enteroviruses. *EV-B*, *EV-C, EV-D*, *RV-A*, *RV-B* and *RV-C* consist of 63, 23, 5, 80, 32 and 56 types respectively^1–3^ (http://www.picornaviridae.com/enterovirus/enterovirus.htm).

All rhinoviruses infect the human respiratory tract, usually limited to the upper respiratory airways, causing the common cold (generally a benign and self-limited illness) both in children and adults^1,4,5^. In contrast, enteroviruses primarily infect the human gastrointestinal tract and can spread to other organs, such as eye, heart or the central nervous system. Although most enterovirus infections are asymptomatic, but over 20 clinically recognized syndromes, including poliomyelitis, encephalitis, meningitis, hand foot and mouth disease (HFMD), myocarditis, respiratory illnesses, febrile illnesses, pleurodynia, herpangina, conjunctivitis, gastroenteritis, myopericarditis, hepatitis and pancreatitis, have been frequently associated with the 103 EV types^1,6^. Among these diseases, HFMD is a global and the most common disease that usually affects infants and children, particularly in those younger than 5 years of age, and can even cause death in some cases^7–9^.

Molecular epidemiological studies have shown that EVs, including *RV-A*, *RV-B* and *RV-C*, are prevalent temporally and geographically in both patients and healthy individuals^10–14^. Consistently, seroepidemiological studies of neutralizing antibodies (Ntb) against EV71 and CA16 demonstrated the prevalence of infections in individuals of all ages. In particular, Ntb against both EV71 and CA16 showed a similar age-related dynamic change among infants and children: neutralizing antibodies in approximately half of the neonates (50–57.6%), obtained from their mothers, are lost within 6–7 months of age, then gradually increase from 7–12 months of age with the occurrence of the virus infections and eventually reach a peak level (above 80–100%) in children usually 4 to 5 years of age, maintaining a high seroprevalence (40–85.3%) in adults^15–20^. All of this evidence further demonstrated the ubiquitous infections by various species of EVs in humans. Our previous study revealed that the anti-EV71 non-neutralizing antibody response was predominantly against VP1, particularly against the epitopes based on the common enterovirus cross-reactive sequence in VP1^21^, and proposed that the non-neutralizing antibody response represents the major host antibody response to EV71 infection and could highly cross-react with the EV71-related viruses, whereas the neutralizing antibody response represents the minor antibody response, which shows high specificity and little cross-reactivity. The nature of the non-neutralizing antibody responses among infants and children remains unknown. In the present study, the non-neutralizing antibody responses against VP1 of EV71 and CA16 in *EV-A*, CB3 in *EV-B*, PV1 in *EV-C* and RV13 in *RV-A* among infants and children aged 1 day to 6 years (1d–6y) in Shanghai were assessed and analyzed. The results identified an age-related dynamic change of antibody responses against VP1 of all of the assessed EVs, revealed the maternally-derived antibody responses and primary antibody responses and demonstrated the different levels of anti-VP1 for various EVs (*EV-A, EV-B, EV-C* and *RV-C*) among infants and children, and could help further understand the ubiquity of EV infections.

## Results

### Expression and Purification of various recombinant VP1 proteins

**Figure 1:**
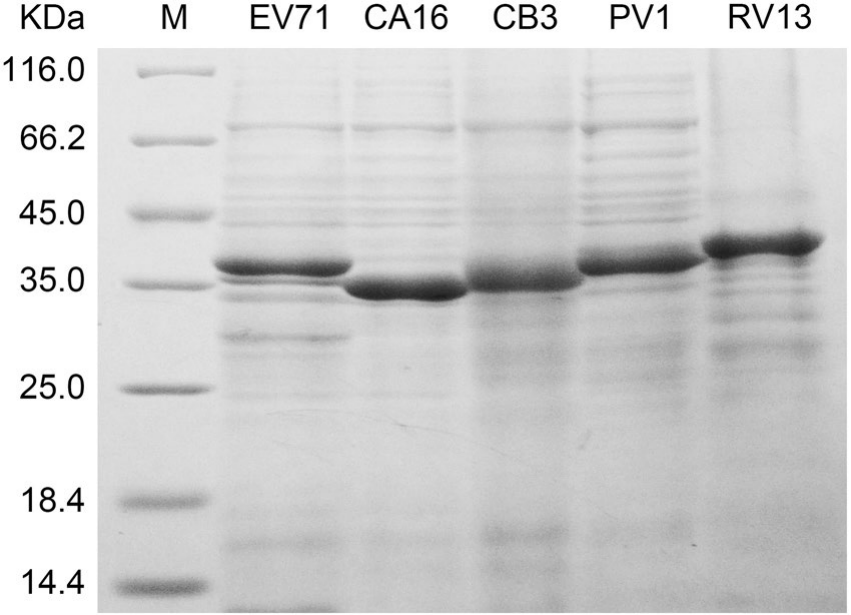
Expression and purification of recombinant EV71, CA16, CB3, PV1 and RV13 VP1 proteins. The relative molecular weights (MW) of the fusion proteins were 35,636 for pET21b-EV71 VP1, 34,242 for pET21b-CA16 VP1, 34,797 for pET21b-CB3 VP1, 34,902 for pET21b-PV1 VP1, 36,192 for pET21b-RV13 VP1. The expressed proteins were purified using Ni-NTA column affinity chromatography.

In our previous study, the antibody response against EV71 capsid proteins was found to predominantly target VP1^21^. In the current study, the VP1 of various EVs from four species of the *Enterovirus* genus (EV71 and CA16 from *EV-A*, CB3 from *EV-B*, PV1 from *EV-C*, and RV13 from *RV-A)* were separately amplified and inserted into prokaryotic expression vector (pET21b). The cloned transformants were subsequently expressed with IPTG in *E. coli* and investigated by SDS-PAGE (Fig. 1). The size of each recombinant protein was in agreement with the expected molecular weight. All His-tag fusion proteins were located in inclusion bodies. These proteins were insoluble, but they could be solubilized in 8 M urea and purified under denaturing conditions.

### Non-neutralizing antibody responses against VP1 of *EV-A*, *EV-B*, *EV-C* and *RV-A* among infants and children in Shanghai

**Figure 2:**
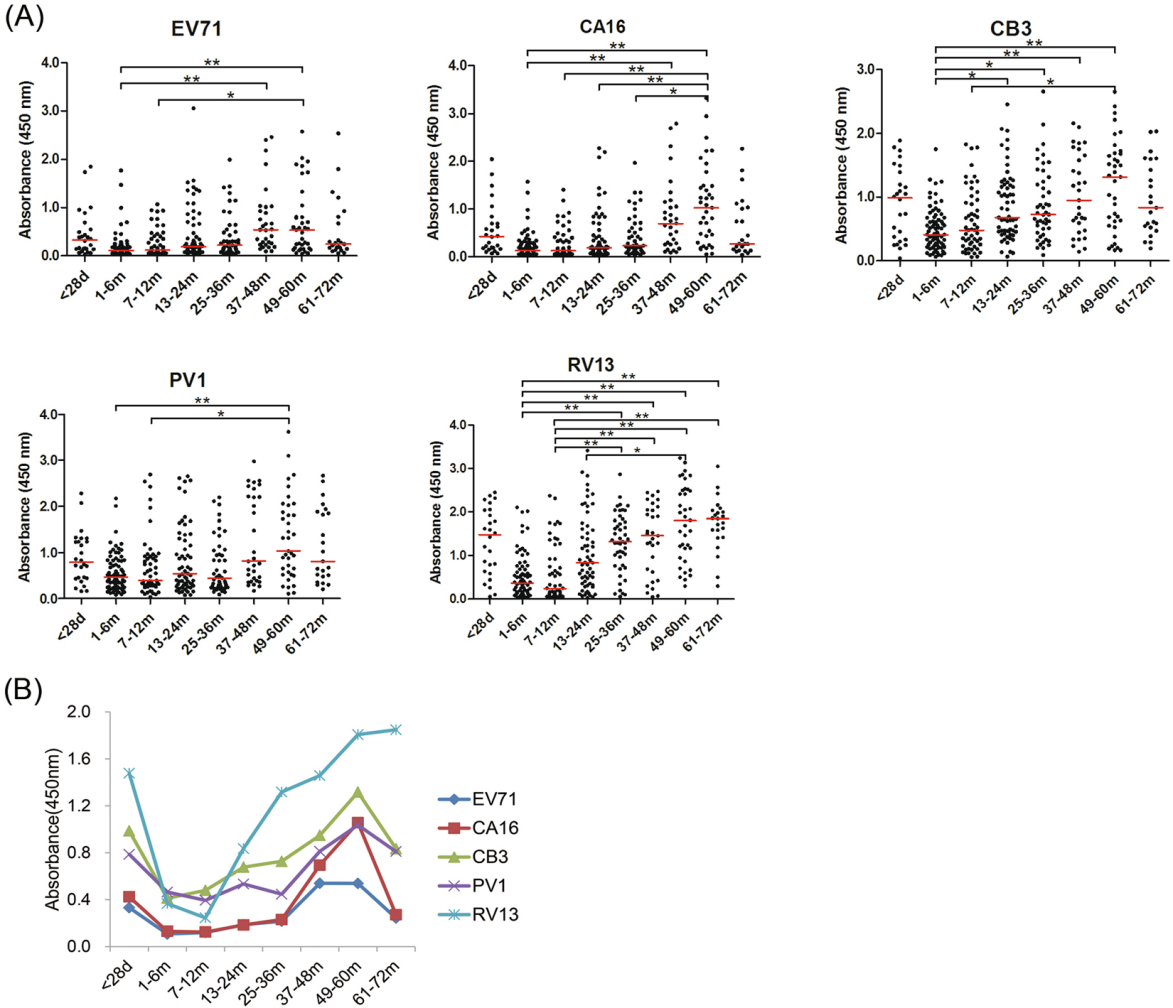
Characteristics of the antibody reactivity against VP1 of EV71, CA16, CB3, PV1 and RV13 in the serum samples of infants and children from eight age groups (1–28-day, 1–6-month, 7–12-month, 13–24-month, 25–36-month, 37–48-month, 49–60-month and 61–72-month age groups). (**A**) Comparison of the anti-EV71, anti-CA16, anti-CB3, anti-PV1 or anti-RV13 VP1 reactivity in eight age groups. Each symbol represents an individual sample, and the line represents the median of the samples. Statistical significance was tested using the Nemenyi non-parametric test. * indicates p < 0.05, ** indicates p < 0.001. (**B**) The age-related dynamic change of antibody reactivity against VP1 of EV71, CA16, CB3, PV1 and RV13 in eight age groups.

**Figure 3:**
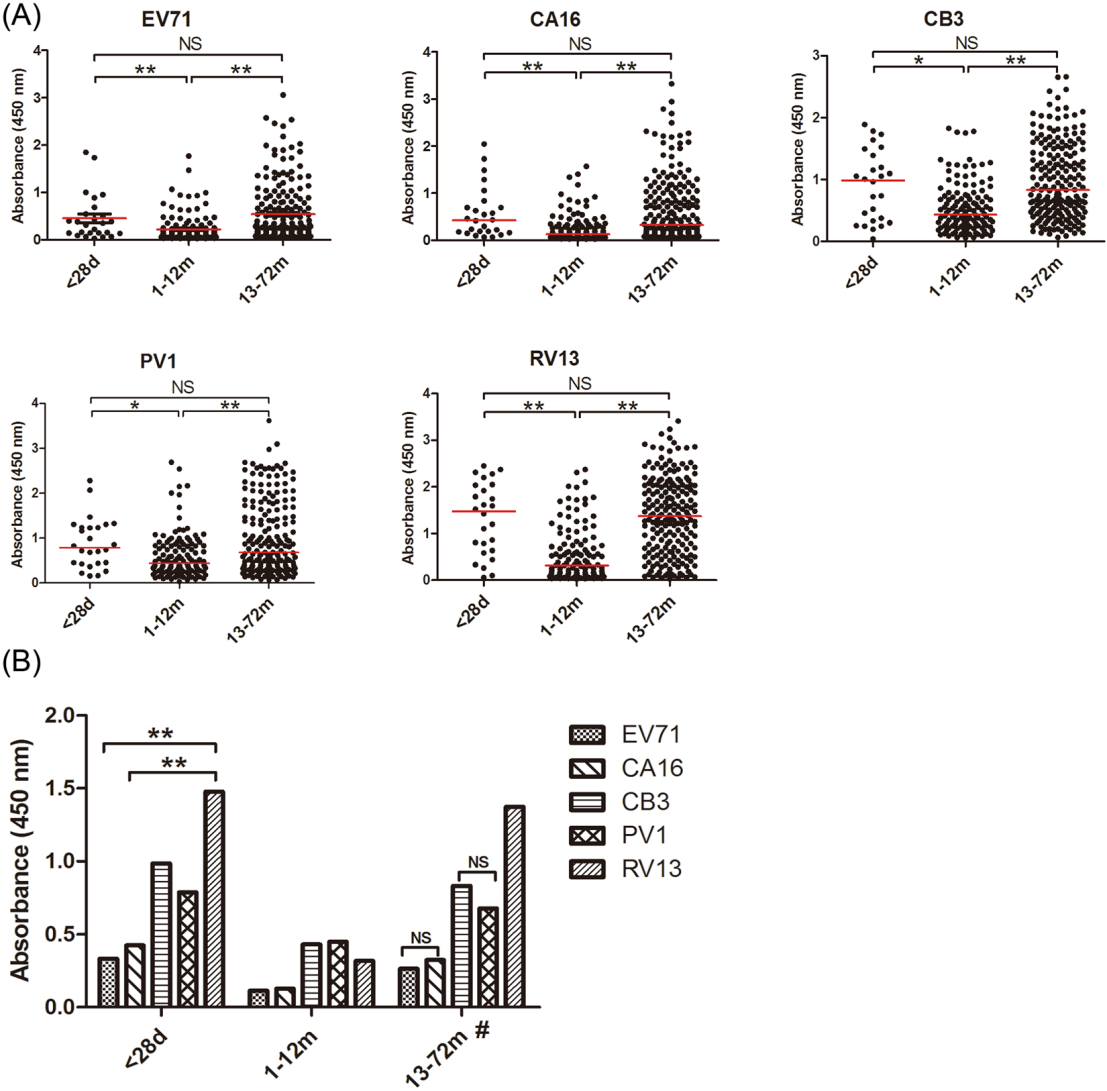
Characteristics of the antibody reactivity against VP1 of EV71, CA16, CB3, PV1 and RV13 in the serum samples from three age groups (1–28-day, 1–12-month and 13–72-month age groups). (**A**) Comparison of the antibody reactivity against VP1 of EV71, CA16, CB3, PV1 and RV13 among three age groups. Each symbol represents an individual sample. The line represents the median of the samples. (**B**) Comparison of the median of anti-VP1 reactivity of the five assessed EVs among three age groups. Statistical significance was tested using the Nemenyi non-parametric test or one-way ANOVA analysis. * indicates p < 0.05, ** indicates p < 0.001, “NS” represents no significant difference between the two groups (p > 0.05). In the 13–72-month age group, ^#^ represents p < 0.05 between the antibody reactivity against any two VP1 from the five assessed EV VP1 proteins except two pairs: EV71 vs CA16 and CB3 vs PV1.

In total, 364 serum samples of infants and children from eight age groups aged 1d-6y collected from Shanghai were utilized to perform NEIBM-ELISA to determine the non-neutralizing antibody responses against VP1 of EV71 and CA16 from *EV-A*, CB3 from *EV-B*, PV1 from *EV-C*, and RV13 from *RV-A*. As shown in Fig. 2, the anti-VP1 reactivity changed similarly in an age-related dynamic: being high level in the 1–28-day age group (27 samples), declining to the lowest level in the 1–12-month of age group (134 samples), gradually increasing to the peak level in the 13–60-month age group (180 samples), then remarkably declining in the 61–72-month age group (23 samples) except for RV13. Based on this finding, we proposed that the antibody responses in the 1–28-day age group represent the maternally-derived antibody responses, the antibody responses in the 13–72-month age group represent the primary antibody responses, and the antibody responses in the 1–12-month age group represent the conversion of maternally-derived antibody responses to primary antibody responses. The antibody responses in these age groups were further analyzed. As shown in Fig. 3A,the anti-VP1 reactivity of all assessed EVs in the 1–28-day age group showed the same high levels as those in the 13–72-month age group, which were significantly higher than those in the 1–12-month age group. The anti-VP1 reactivity in the 1–12-month age group was the lowest in three age groups (Fig. 3A), and the antibody responses in this age group might be mixed with maternally-derived and primary antibody responses and will complicate our analysis. Therefore, only the anti-VP1 reactivity in 1–28-day and 13–72-month age groups was compared and further analyzed. As shown in Fig. 3B, the anti-RV13 VP1 reactivity in the 1–28-day age group was significantly higher than the reactivity against VP1 of EV71 and CA16 and demonstrated the highest level, with the antibody reactivity against VP1 of CB3 and PV1 at the second highest level, and the antibody reactivity against VP1 of EV71 and CA16 at the lowest level. Similarly, the antibody reactivity against RV13 VP1 in the 13–72-month age group was significantly higher than that against the other assessed EVs and demonstrated the highest level, with the antibody reactivity against VP1 of CB3 and PV1 were significantly higher than those against VP1 of EV71 and CA16 and showed the second highest level, and the antibody reactivity against VP1 of EV71 and CA16 showed the lowest level. It is interesting that the order of anti-VP1 reactivity levels of all assessed EVs from high to low (RV13 > CB3 > PV1 > CA16 > EV71) in the 1–28-day age group was the same as that in 13–72-month age group. These results consistently suggested that the antibody responses in the 1–28-day age group likely represent the maternally-derived antibody responses and that the antibody responses in the 13–72-month age group represent primary antibody responses.

**Figure 4:**
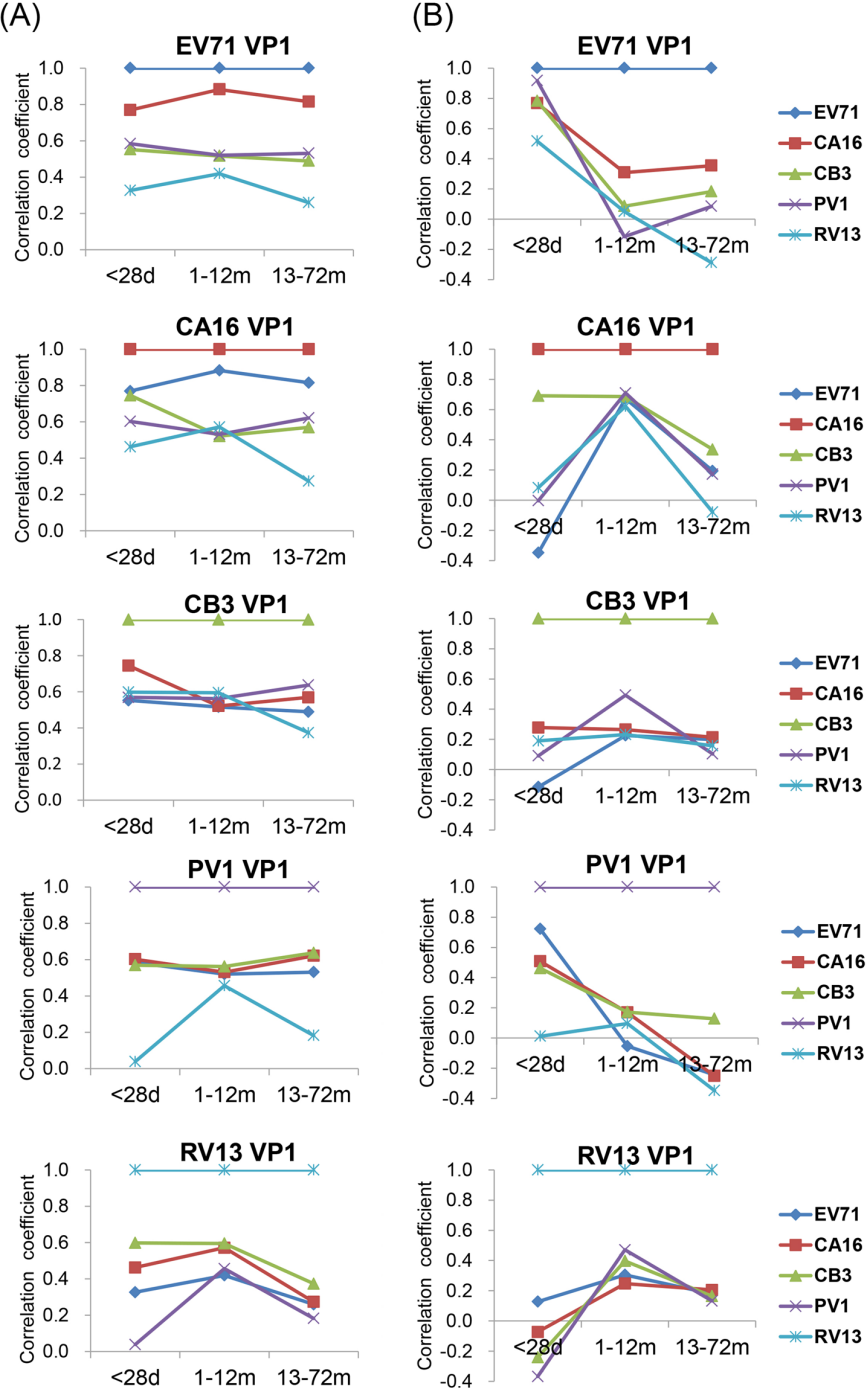
Comparison of the correlation analyses between five assessed EV VP1 proteins based on antibody responses or inhibition to anti-VP1 reactions by various EV VP1 proteins in three age groups (1–28-day, 1–12-month and 13–72-month age groups). (**A**) Correlation analysis of antibody reactivity against VP1 of EV71, CA16, CB3, PV1 and RV13 in three age groups. (**B**) Correlation analysis of inhibition to the anti-VP1 reactions of five assessed EVs, EV71, CA16, CB3, PV1 or RV13 by various VP1 proteins in three age groups.

To further investigate the antibody responses against these EVs, a correlation analysis of the antibody reactions in the 1–28-day and 13–72-month age groups was performed. As shown in Table 1 and Fig. 4A, in the 1–28-day age group, the correlations between the reactions against VP1 of EV71 and CA16 in the *EV-A* species in both groups demonstrated the highest levels, with correlation coefficients of approximately 0.8; the correlations between reactions against VP1 of EVs in *EV-A*, *EV-B* or *EV-C* species, which infect the human gastrointestinal tract, demonstrated the second highest levels, with correlation coefficients from 0.5 to 0.7; and the correlations between reactions against VP1 of RV13 in the *RV-A* species, which infect the human respiratory tract, and VP1 of the EVs in the *EV-A*, *EV-B* or *EV-C* species demonstrated the lowest levels, with correlation coefficients less than 0.5. These results suggested that the correlation level between reactions against VP1 of various EV species was highly associated with the phylogenetic classification of EV species. No significant change in correlation coefficients of reactions against VP1 of EVs between the 1–28-day and 13–72-month age groups was observed.

**Table 1:** The correlation coefficients of anti-VP1 reactions between five enteroviruses, EV71, CA16, CB3, PV1 and RV13 among infants and children from three age groups (1–28-day, 1–12-month and 13–72-month age groups).

		EV71	CA16	CB3	PV1	RV13
EV71	<28d	—	0.769^**^	0.552^**^	0.584^**^	0.326
	1–12 m	—	0.883^**^	0.516^**^	0.520^**^	0.419^**^
	13–72 m	—	0.815^**^	0.489^**^	0.531^**^	0.259^**^
CA16	<28d		—	0.745^**^	0.602^**^	0.462*
	1–12 m		—	0.521^**^	0.531^**^	0.571^**^
	13–72 m		—	0.569^**^	0.621^**^	0.273^**^
CB3	<28d			—	0.569^**^	0.598^**^
	1–12 m			—	0.562^**^	0.595^**^
	13–72 m			—	0.637^**^	0.373^**^
PV1	<28d				—	0.038
	1–12 m				—	0.456^**^
	13–72 m				—	0.182^**^

* represents p < 0.05. ** represents p < 0.001.

### A competitive inhibition ELISA demonstrated different antibody responses against VP1 of the *EV-A*, *EV-B*, *EV-C* and *RV-A* species between the 1–28-day and 13–72-month age groups

**Figure 5:**
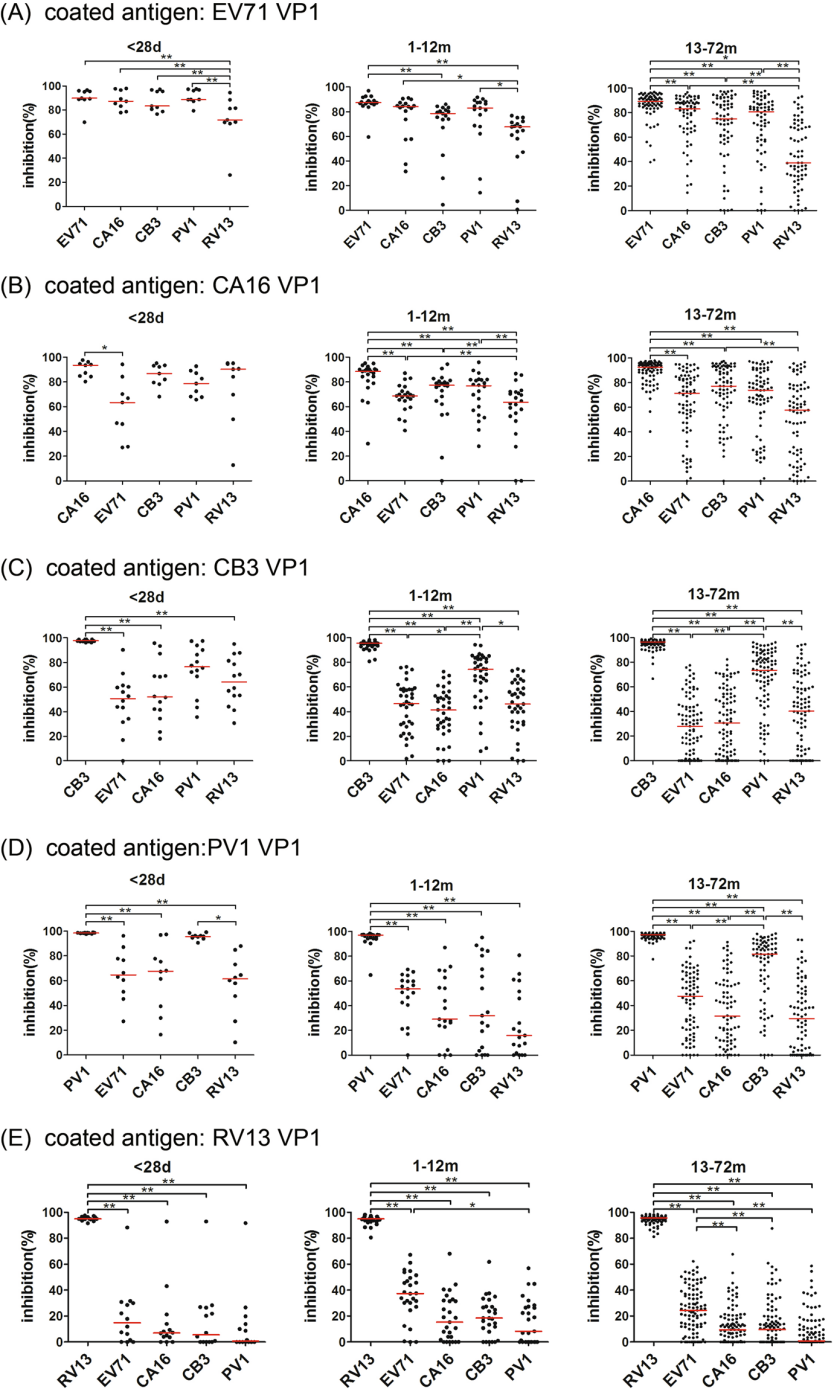
Comparison of inhibition activities to anti-VP1 reactions of EV71 (**A**), CA16 (**B**), CB3 (**C**), PV1 (**D**), and RV13 (**E**) by five EV VP1 proteins in the 1–28-day, 1–12-month and 13–72-month age groups. The coated antigen was EV71 VP1 (**A**), CA16 VP1 (**B**), CB3 VP1 (**C**), PV1 VP1 (**D**) and RV13 VP1 (**E**) respectively. The percent inhibition from the competitive ELISA was plotted on the y-axis with the five inhibitor proteins on the x-axis: VP1 of EV71, CA16, CB3, PV1 and RV13. The line represents the median of the samples. Statistical significance was tested using the Nemenyi non-parametric test. * represents p < 0.05, ** represents p < 0.001.

To further investigate the non-neutralizing antibody response in detail, 92 samples (9 from the 1–28-day age group, 17 from the 1–12-month age group and 66 from the 13–72-month age group) that were strongly reactive against EV71 VP1 were analyzed in a competitive inhibition ELISA. Also, 106 (9, 22 and 75 from three age groups), 142 (14, 37, 91 from three age groups), 103 (10, 19 and 74 from three age groups) and 136 (14, 27 and 95 from three age groups) serum samples that were strongly reactive against VP1 of CA16, CB3, PV1 and RV13 were chosen respectively to further assess the antibody reactions against VP1 of CA16, CB3, PV1 and RV13 in a competitive ELISA as conducted above. As shown in Fig. 5A, when EV71 VP1 was coated, five EV VP1 proteins, EV71, CA16, CB3, PV1 and RV13 VP1, were utilized to inhibit the anti-EV71 VP1 reaction. The anti-EV71 VP1 reactivity was completely inhibited by EV71 VP1 (inhibition rate > 80%) in both the 1–28-day and the 13–72-month age groups and effectively inhibited by the VP1 of three other EVs, CA16, CB3 and PV1 in these two groups (inhibition rate between 70% and 80%). Similarly, the anti-CA16 VP1 reactivity was completely inhibited by CA16 VP1 in both the 1–28-day and the 13–72-month age groups, and effectively inhibited by VP1 of three other EVs, CB3, PV1 and RV13 in the 1–28-day age group and by VP1 of three other EVs, EV71, CB3 and PV1 in the 13–72-month age group (Fig. 5B). Differently, the anti-CB3 VP1 reactivity was completely inhibited by CB3 VP1 in both the 1–28-day and 13–72-month age groups, but effectively inhibited only by PV1 VP1(Fig. 5C). The anti-PV1 VP1 reactivity was completely inhibited by PV1 VP1 in both the 1–28-day and the 13–72-month age groups, and effectively inhibited only by CB3 VP1 (Fig. 5D). Of note, the anti-RV13 VP1 reactivity was only completely inhibited by RV13 VP1 in both the 1–28-day and the 13–72-month age groups, and was not effectively inhibited by the VP1 of the other assessed EVs (Fig. 5E). These results further demonstrated that the reactivity level of anti-RV13 VP1 was the highest, the reactivity levels of anti-VP1 of CB3 and PV1 were the second highest, and the reactivity levels of anti-VP1 of CA16 and EV71 were the lowest.

Importantly, many significant differences in antibody responses between the 13–72-month and the 1–28-day age groups were observed. As shown in Fig. 5, for the samples from the 13–72-month age group, the VP1 itself of each EV exhibited a significantly stronger inhibitory potency to the anti-VP1 reactions than any VP1 from other EVs. In contrast, in the samples from the 1–28-day age group, the VP1 of CA16, CB3 and PV1 showed the same strong inhibitory potency to anti-EV71 VP1 as did EV71 VP1, implying that the anti-EV71 VP1 antibodies in this age group contained more nonspecific antibodies from CA16, CB3 and PV1 infections compared with those in 13–72-month age group. Similarly, the VP1 of CB3, PV1 and RV13 showed the same strong inhibitory potency to anti-CA16 VP1 reactivity as did CA16 VP1 in the 1–28-day age group, implying that anti-CA16 VP1 antibodies contained more nonspecific antibodies from CB3, PV1 and RV13 infections in this group. PV1 VP1 showed the same strong inhibitory potency to anti-CB3 VP1 reactivity as did CB3 VP1 in the 1–28-day age group, implying that anti-CB3 VP1 antibodies in this age group contained more nonspecific antibodies from PV1 infection. CB3 VP1 showed the same strong inhibitory potency to anti-PV1 VP1 reactivity as did PV1 VP1, implying that anti-PV1 VP1 antibodies in the 1–28-day age group contained more nonspecific antibodies from CB3 infection. Exceptionally, the RV13 VP1 showed a significantly stronger potency than all of the VP1 from the other EVs in the 1–28-day age group, implying that the antibody response against RV13 VP1 was not significantly affected by the cross-reactive antibodies from the infections by the other EVs. These results indicated the antibody responses against VP1 of EV71, CA16, CB3 and PV1, which usually infect the human gastrointestinal tract, contained more nonspecific antibodies and less specific antibodies in the samples from the 1–28-day age group than those from the 13–72-month age group.

**Figure 6:**
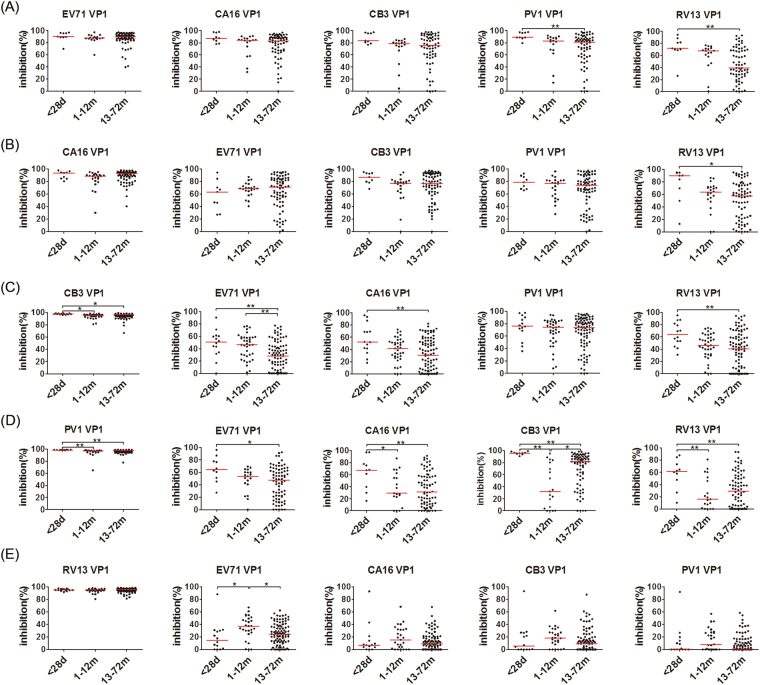
Comparison of inhibitory potencies to anti-VP1 reactions of EV71 (**A**), CA16 (**B**), CB3 (**C**), PV1 (**D**), and RV13 (**E**) in three age groups by five EV VP1 proteins, EV71, CA16, CB3, PV1 and RV13 VP1. The coated antigen was EV71 VP1 (**A**), CA16 VP1 (**B**), CB3 VP1 (**C**), PV1 VP1 (**D**) and RV13 VP1 (**E**), respectively. The inhibitory protein had been marked above the figure. The percent inhibition from the competitive ELISA was plotted on the y-axis with three age groups on the x-axis. The line represents the median of the samples. Statistical significance was tested using the Nemenyi non-parametric test. * represents p < 0.05, ** represents p < 0.001.

Moreover, as shown in Fig. 6, some VP1 showed a significantly different inhibitory potency to the anti-VP1 reactions of all assessed EVs (except for RV13) between the samples from the 1–28-day and the 13–72-month age groups: the VP1 of RV13 and PV1 showed the significantly stronger inhibitory potency to anti-EV71 VP1 reactivity in the samples from the 1–28-day age group than those from the 13–72-month age group, thus demonstrating the anti-EV71 VP1 antibodies in the samples from the 1–28-day age group contained more nonspecific antibodies from RV13 and PV1 infections or their related EVs than did those from the 13–72-month age group; the RV13 VP1 showed a significantly stronger inhibitory potency to anti-CA16 VP1 reactivity in the samples from the 1–28-day age group than those from the 13–72-month age group, demonstrating that the anti-CA16 VP1 antibodies in the 1–28-day age group contained more nonspecific antibodies from RV13 infection or its related EVs; the VP1 of EV71, CA16 and RV13 showed a significantly stronger inhibitory potency to anti-CB3 VP1 reactivity in the samples from the 1–28-day age group than those from the 13–72-month age group, demonstrating that the anti-CB3 VP1 antibodies in the 1–28-day age group contained more nonspecific antibodies from EV71, CA16, RV13 infections or their related EVs; the VP1 of EV71, CA16, CB3 and RV13 showed significantly stronger inhibitory potencies to anti-PV1 VP1 reactivity in the samples from the 1–28-day age group than those from the 13–72-month age group, demonstrating that the anti-PV1 VP1 antibodies in the 1–28-day age group contained more nonspecific antibodies from EV71, CA16, CB3 and RV13 infections or their related EVs. Of note, none of the VP1 showed a significant difference in inhibitory potency to anti-RV13 VP1 reactivity between the samples from the 1–28-day and the 13–72-month age groups. These results also demonstrated that the antibody responses against VP1 of EV71, CA16, CB3 and PV1 in the samples from the 1–28-day age group contained more non-specific antibodies from infections by other EVs than those in the 13–72-month age group.

A correlation analysis of the inhibition of the various anti-VP1 reactions was also performed to characterize the antibody responses between the 1–28-day and the 13–72-month age groups. In contrast to the results of the correlation of the anti-VP1 reactions, as shown in Table 2 and Fig. 4B, an obvious change in the correlation coefficients for inhibition was observed in many cases between the 1–28-day age group and the 13–72-month age group. The correlation coefficients for inhibition of anti-EV71 VP1 reactivity by EV71 VP1 and by VP1 of CA16, CB3, PV1 or RV13 in the samples from the 1–28-day age group were 0.767, 0.783, 0.917 and 0.517, respectively, while the equivalent correlation coefficients in the samples from the 13–72-month age group were 0.354, 0.183, 0.085 and −0.288, respectively, indicating that the anti-EV71 VP1 antibodies in the samples from the 1–28-day age group contained more nonspecific antibodies from infections by CA16, CB3, PV1 and RV13 or their related EVs than that did the 13–72-month age group. Similarly, the correlation coefficients for inhibition of anti-PV1 VP1 reactivity by PV1 VP1 and by VP1 of EV71, CA16 or CB3 in the samples from the 1–28-day age group were 0.722, 0.508 and 0.462, respectively, while the equivalents in the samples from the 13–72-month age group were −0.244, −0.251 and 0.128, respectively, indicating that the anti-PV1 VP1 antibodies in the samples from the 1–28-day age group contained more nonspecific antibodies from infections by EV71, CA16 and CB3 or their related EVs than did the 13–72-month age group. The correlation coefficients for inhibition of anti-CA16 VP1 reactivity by CA16 VP1 and CB3 VP1 in the samples from the 1–28-day age group was 0.691, while the equivalent in the 13–72-month age group was 0.336, likely indicating the anti-CA16 VP1 antibodies in the samples from the 1–28-day age group contained more nonspecific antibodies from infection by CB3 or its related EVs than did the 13–72-month age group. In contrast, the correlation coefficients for inhibition of anti-CB3 VP1 reactivity by CB3 VP1 and the VP1 of other EVs in the samples from the 1–28-day and the 13–72-month age groups were less than 0.300 and showed no obviously change. These results consistently demonstrated that the antibody responses against VP1 of EV71, CA16 and PV1 contain more non-specific antibodies elicited by infections by other EVs in the samples from the 1–28-day age group than do the samples from the 13–72-month age group.

**Table 2:** The correlation coefficients of inhibition to anti-VP1 reactions of five enteroviruses, EV71, CA16, CB3, PV1 and RV13 by the five EV VP1 proteins among infants and children from three age groups (1–28-day, 1–12-month and 13–72-month age groups).

Coated antigens^#^	Age group	Inhibition proteins				
		EV71	CA16	CB3	PV1	RV13
EV71^#^	<28d	—	0.767^*^	0.783^*^	0.917^**^	0.517
	1–12 m	—	0.309	0.086	−0.115	0.051
	13–72 m	—	0.354^**^	0.183	0.085	−0.288*
CA16^#^	<28d	−0.348	—	0.691^*^	−0.003	0.083
	1–12 m	0.673^**^	—	0.686^**^	0.710^**^	0.622^**^
	13–72 m	0.194	—	0.336^**^	0.172	−0.079
CB3^#^	<28d	−0.115	0.278	—	0.091	0.190
	1–12 m	0.226	0.265	—	0.493^**^	0.232
	13–72 m	0.199	0.214^*^	—	0.104	0.158
PV1^#^	<28d	0.722^*^	0.508	0.462	—	0.012
	1–12 m	−0.054	0.169	0.172	—	0.096
	13–72 m	−0.244^*^	−0.251^*^	0.128	—	−0.348^**^
RV13^#^	<28d	0.128	−0.073	−0.241	−0.368	—
	1–12 m	0.305	0.247	0.397^*^	0.471^*^	—
	13–72 m	0.179	0.205^*^	0.164	0.132	—

* represents p < 0.05. ** represents p < 0.001. ^#^These coated antigens were also used as inhibition proteins.

## Discussion

The neutralizing antibody assay has been commonly used for the serological detection and surveillance of EV infections. However, this assay is labor-intensive, time-consuming and difficult to conduct on a large-scale detection, requiring using viable viruses, specialized equipment and trained personnel. Moreover, it cannot provide overall information on EV infections in human. In this study, we used a NEIBM-based ELISA to study the non-neutralizing antibody responses against the VP1 of EV71, CA16, CB3, PV1 and RV13 among infants and children aged 1d-6y in Shanghai. Very interestingly, the non-neutralizing antibody responses against the VP1 of all assessed EVs showed an age-related dynamic change, which were similar to that of the neutralizing antibody responses: present at the high levels in the 1–28-day age group, declining to the lowest level in the 1–12-month age group, gradually increasing to the peak level during 13–60-month age group, then markedly declining in the 61–72-month age group (except for RV13). Based on this finding, we proposed that the higher level of antibodies in the 1–28-day age group compared to those in the 1–12-month age group were likely to be the maternally-derived antibodies, which usually declined greatly in 6 months of age and diminished in 1 year of age, and the higher level of antibodies in the 13–60-month age group were likely elicited by primary EV infections. With the accumulation of primary infection, the antibody level gradually increased from the 7–12-month age group and peaked in the 49–60-month age group. The remarkable decline in the antibody level against VP1 of EV71, CA16, CB3 and PV1 in the 61–72-month age group likely indicates the end of the primary infection. This finding could well explain the fact that the HFMD predominantly occurred in children under 5 years of age, especially those less than 3 years old. Of note, the antibody level against VP1 of RV13, which infects the human respiratory tract, did not decline and maintained a peak level in the 61–72- month age group, which might be because of the prolonged primary infection by other rhinoviruses.

Consistently, the neutralizing antibody responses against EV71 and CA16 also showed a similar age-related dynamic change among infants and children^15–20,22,23^. Intriguingly, some obvious differences between the age-related dynamic change of the non-neutralizing antibody responses and the neutralizing antibody responses against EV71 or CA16 were observed. The first difference is that the seroprevalence rates of the neutralizing antibodies and the geometric mean titers (GMT) against both EV71 and CA16 reached a peak level at 4 years of age, 1 year prior to the non-neutralizing antibodies in the present study. Our explanation for this discrepancy is that the non-neutralizing antibodies detected in this study contain both specific antibodies and cross-reactive antibodies. The cross-reactive antibodies which were elicited by the infections with many other related EVs could contribute to the continuing increase in antibody level after the peak of specific antibodies and to the development of the delayed non-neutralizing antibodies’ peak. The second difference is unlike the high level of anti-CA16 non-neutralizing antibody in the 1–28-day age group, which is significantly higher than that in 1–12-month age group, anti-CA16 neutralizing antibody in the 1–28-day age group didn’t usually show the higher level than that in the 1–12-month age group^15^. This was probably because of low exposure to CA16 in their mothers, both the seroprevalence rates of the neutralizing antibodies and the GMT against CA16 in the 1–28-day age group were in a very low level^15,16,20^. Besides, the technical limitation for detecting the neutralizing antibodies against CA16 in the assay might be one of the reasons. The third obvious difference is that the antibody level of the non-neutralizing antibodies against all assessed EVs in *EV-A, B, C* species declined markedly after the peak occurred, which could indicate the end of primary EV infections, but was not always clearly observed in the neutralizing antibodies against EV71 and CA16^15–20,22,23^. All these differences could demonstrate the distinctive significance of non-neutralizing antibody assay. Taken together, our results indicated the primary EV infections among infants and children and demonstrated that the non-neutralizing antibody assay could provide comprehensive, accurate and valuable information on the prevalence of infections by EVs, which could complement the neutralizing antibody assay and become a novel useful method for the serological study of EV infections.

Our results demonstrated that the anti-RV13 VP1 antibodies in both the 1–28-day and the 13–72-month age group were at the highest level, with the anti-VP1 of CB3 and PV1 antibodies at the second highest level and the anti-VP1 of EV71 and CA16 antibodies at the lowest level. This result suggested that the levels of the antibody responses against VP1 of RV13 in *RV-A*, CB3 in *EV-B*, PV1 in *EV-C*, and EV71 and CA16 in *EV-A* seemed to be generally proportional to the numbers of isolated types or serotypes in the three species: 80 types in *RV-A*, 63 types in *EV-B*, 23 types in *EV-C* and 25 *types* in EV-A. This phenomenon was consistent with our previous finding in Shanghai blood donors^24^ and still needed to be further confirmed. In the present study, the antibodies of anti-VP1 of PV1 in *EV-C*, which contains 23 types, showed a higher level compared with those of anti-VP1 of EV71 and CA16 in *EV-A*, which contains 25 types. This could result from the regular vaccination of infants at 2, 3, 4 months of age and children at 4 years of age with the attenuated live polio vaccine in China.

In this study, the peak antibody level of anti-VP1 of EV71 in the 49–60-month age group was lower compared with that of CA16. However, the peak seroprevalence rates of the EV71 neutralizing antibody among children aged 4 years in China was about 90%^15^, suggesting that nearly the entire population of children had been infected by EV71 before 4 years of age, while the peak seroprevalence rates of the CA16 neutralizing antibody among children aged 4 years was approximately 70%, which is less than that of EV71^15^. In contrast, our results showed that the non-neutralizing antibody level against CA16 VP1 seemed higher than that against EV71 VP1, which was not consistent with the peak seroprevalence rates in the neutralizing antibody assay. Based on these findings, we favour the idea that nearly the entire population of children had been primarily infected by various EVs within 1–5 years of age and that the levels of anti-VP1 reactivity of the assessed EVs from various species were likely related to the numbers of isolated types or serotypes in their species but not to the seroprevalence rate.

Very interestingly, our results from the competitive inhibition ELISA demonstrated different antibody responses against VP1 of EVs between the 1–28-day and the 13–72-month age groups. In theory, the antibody responses against VP1 in the 13–72-month age group represented the antibody responses of the primary infections by EVs. Whereas the antibody responses against VP1 in the 1–28-day age group represented the IgG antibody responses transferred from their mothers, which were elicited mainly in response to the re-infections by various EVs, and should become focused on the CECRS-based epitopes, likely cross-reacting with the VP1 of other EVs^21^. Therefore, the antibodies in the 1–28-day age group would contain a higher quantity of CECRS-based cross-reactive antibodies and a lower quantity of specific antibodies than would the antibodies in the 13–72-month age group, in which the specific antibodies were produced mainly in response to primary infections by EVs. The results of the competitive inhibition ELISA demonstrate this assumption. This assumption was particularly obvious in the antibody responses against PV1 VP1. In China, the last indigenous poliomyelitis case was reported in 1994, and the country was certified as polio-free in 2009^25^. In fact, the anti-PV1 VP1 reactivity in the samples from the 1–28-day age group represented the maternally-derived IgG antibody responses and must be non-specific antibodies. In contrast, the anti-PV1 VP1 reactivity in the samples from the 13–72-month age group were likely generated by vaccination with attenuated live polio vaccine at 2, 3 and 4 months and at 4 years of age respectively, and could represent the specific antibody responses. Consistently, the VP1 of all assessed EVs (EV71, CA16, CB3 and RV13) showed a significantly stronger inhibitory potency to anti-PV1 VP1 reactivity in the samples from the 1–28-day age group than did those from the 13–72-month age group (Fig. 6D), and the correlation coefficients for the inhibition of anti-PV1 VP1 reactivity by PV1 VP1 and VP1 of all the assessed EVs other than PV1 in the samples from the 1–28-day age group were obviously higher than those in the samples from the 13–72-month age group (Table 2 and Fig. 4B), clearly discriminating between the non-specific antibody responses from the infections by other related EVs in the 1–28-day age group and the specific antibody responses from the primary infection by PV1 in the 13–72-month age group. These results clearly demonstrate the nature of the cross-reactive antibody responses in the 1–28-day age group and the primary antibody responses in 13–72-month age group.

This study is the first to describe the overall non-neutralizing antibody responses against VP1 of the EVs in *EV-A, B, C* and *RV-A* species among infants and children. The age-related dynamic change of the non-neutralizing antibody responses against VP1 of the EVs were revealed, the maternally-derived antibody responses and primary antibody responses were characterized, and the different levels of antibody responses against various EVs were demonstrated. These findings might be helpful for further understanding of ubiquitous EV infections, especially among infants and children, and could also provide a novel useful approach for the serological study of EV infections.

## Materials and Methods

### Ethical statement

This study was approved by the Ethics Committee of the Children’s Hospital of Fudan University, Shanghai, China. All information and patient identifiers were kept anonymous to protect patient confidentiality. All experiments were performed in accordance with the approved guidelines of the Ethics Committee of the Second Military Medical University and Ethics Committee of the Children’s Hospital of Fudan University, Shanghai, China. The written informed consent was provided by all participants in the study.

### Clinical samples

Three hundred and sixty-four serum specimens from infants and children aged 1d-6y were collected from the Children’s Hospital of Fudan University, Shanghai, China from 5 May to 24 June 2015. Relevant information for each of the 364 serum samples was also recorded (Table S1). Eight age groups were classified. There were 27, 80, 54, 62, 47, 33, 38 and 23 serum samples in 1–28-day, 1–6-month, 7–12-month, 13–24-month, 25–36-month, 37–48-month, 49–60-month and 61–72-month age groups, respectively. All samples were aliquoted and stored at −80 °C.

### Vectors, bacterial strains and reagents

The high-efficiency TA cloning vector, pMD18-T, was purchased from TAKARA BIOTECHNOLOGY (DALIAN) CO.,LTD. (Dalian, People’s Republic of China). The prokaryotic expression plasmid pET-21b and two *E. coli* host strains, Top10 and BL21 (DE3), were purchased from Novagen (Darmstadt, Germany). The NEIBM LD5, a novel evolved immunoglobulin-binding molecule with synergistic double binding sites to the VH3 and VƘ regions of the Fab and to the Fc region of IgG^26^, was conjugated with Horseradish peroxidase (HRP-LD5) and used as a labeled secondary antibody. HRP-LD5 shows a high binding affinity for IgM, IgG and IgA^27^.

### Cloning of the gene fragments of the EV71, CA16, CB3, PV1 and RV13 VP1 and construction of the recombinant plasmids

The amino acid sequence of RV13-VP1 (rhinovirus A N13 capsid protein VP1) was obtained from GenBank (GenBank accession number: ACU00187.1). The DNA sequence of RV13 VP1 was synthesized using sequential OE-PCR as previously described^28^ and T/A-cloned into the pMD18-T vector. The previously constructed recombinant EV71 VP1, CA16 VP1, CB3 VP1 and PV1 VP1 plasmids (pMD18-T-EV71 VP1, pMD18-T-CA16 VP1, pMD18-T-CB3 VP1 and pMD18-T-PV1 VP1)^24^ and pMD18-T-RV13 VP1 were used as templates to amplify EV71 VP1, CA16 VP1, CB3 VP1, PV1 VP1 and RV13 VP1 using the primer pairs uEV71/dEV71, uCA16/dCA16, uCB3/dCB3, uPV1/dPV1 and uRV13/dRV13 (Table S2), respectively. The PCR products of EV71 VP1, CA16 VP1, CB3 VP1, PV1 VP1 and RV13 VP1 were digested with different enzymes, purified and inserted into the cloning sites of the prokaryotic expression vector pET21b under the T7 promoter, and a His-tag was added at the C-terminus of the target to generate recombinant plasmids. The recombinant expression plasmids were individually verified by sequencing analysis.

### Prokaryotic expression and purification of recombinant EV71, CA16, CB3, PV1 and RV13 VP1 proteins

*E. coli* BL21 (DE3) competent cells transformed with EV71, CA16, CB3, PV1 and RV13 VP1 expression plasmids (pET-21b-EV71 VP1, pET-21b-CA16 VP1, pET-21b-CB3 VP1, pET-21b-PV1 VP1 and pET-21b-RV13 VP1) were cultured in Luria broth (LB) medium which was supplemented with 100 μg/ml ampicillin at 37 °C. When the optical density value at 600 nm (OD600) of the culture reached 0.6, the cells were induced with Isopropyl β-D-1-thiogalactopyranoside (IPTG) to a final concentration of 1 mM and incubation was continued for 3–4 h. The cells were harvested by centrifugation at 6000 × g for 20 min at 4 °C. Lyse the bacteria pellets by ultrasonication after resuspension in PBS (pH 7.2). Then, the lysates were treated by centrifugation at 11000 × g for 10 min at 4 °C. The inclusion bodies were solubilized in 8 M urea at 4 °C overnight. After centrifugation at 11000 × g for 10 min at 4 °C, the clear supernatant was harvested and used for SDS-PAGE and purification. All the fusion proteins were purified using Ni-NTA resin (Qiagen, Hilden, Germany) and packed to aliquot parts subsequently and stored at −80 °C.

### Indirect ELISA of antibodies against VP1 of various enteroviruses

The antibodies against VP1 of various EVs from four species of the *Enterovirus* genus, EV71 and CA16 from *EV-A*, CB3 from *EV-B*, PV1 from *EV-C* and RV13 from *RV-A* were assessed by ELISA using the NEIBM-derived conjugate HRP-LD5 (NEIBM-ELISA) as previously described^21,29^. Purified EV71, CA16, CB3, PV1 and RV13 VP1 proteins were diluted to a final working concentration (10 μg/ml) in 100 mM carbonate buffer (pH 9.6) and then added to the 96-well microtiter plates (Nunc, Rochester, NY, USA) to a final volume of 100 μl per well. The strips were incubated for 3 h at 37 °C and then blocked with 200 μl of 15% skimmed milk diluted in PBS-Tween 20 (blocking buffer) for 2 h. Next, 100 μl of the serum samples at a dilution of 1:20 were added to each well and then were incubated for 45 min at 37 °C. The strips were washed four times with wash buffer (0.25% Tris base, 0.05% Tween 20), followed by HRP-LD5 (1 mg/ml) at a dilution of 1:2000 and then incubated for 45 min at 37 °C. The reaction was developed by 100 μl 3,3′, 5,5′-tetramethylbenzidine (TMB) (Sigma-Aldrich, St Louis, MO, USA) and hydrogen peroxide mixture and then terminated after suitable color development by 50 μl 2 M sulfuric acid. The optical densities at 450 nm (OD450) was measured using an ELISA Reader (Thermo Scientific Multiskan Fc, Vantaa, Finland).

### Competitive inhibition ELISA

To further investigate the non-neutralizing antibody responses against VP1 of various EVs, a competitive inhibition ELISA was performed as described before^21,30–32^. Purified EV71 VP1 protein was diluted to a final working concentration (10 μg/ml) in 100 mM carbonate buffer (pH 9.6) and then added to the 96-well microtiter plates to a final volume of 100 μl per well. The strips were incubated at 4 °C overnight and then blocked with 200 μl blocking buffer at 37 °C for 2 h. Then, 100 μl of the serum samples at a dilution of 1:20 with high reactivity against EV71 VP1 (the serum samples were sorted by the OD450 value of the reactivity against EV71 VP1, and those with the highest reactivities were selected) from the 1–28-day and 13–72-month age groups first reacted firstly with 2.0 μg of the inhibitor protein (VP1 of EV71, CA16, CB3, PV1 and RV13) at 37 °C for 1 h. Subsequently, the serum in the presence (test serum) and absence (serum control) of inhibitor proteins was added into the strips coated with EV71 VP1 protein and then incubated at 37 °C for 45 min. After washing four times with wash buffer, the strips were added with 100 μl of HRP-LD5 (1 mg/ml) at a dilution of 1:2000 and then were incubated at 37 °C for 45 min. After washing four times, the reaction was developed using a TMB and hydrogen peroxide mixture and stopped by sulfuric acid. The OD450 value was measured using an ELISA Reader. Three parallel wells for each test were conducted, and the mean value was used to determine the percentage of inhibition (PI). The PI was calculated as follows: PI = [100 − (OD450 value of test serum – OD450 value of background)/(OD450 value of serum control – OD450 value of background) × 100)], where the OD450 value of background was obtained in the absence of serum sample or HRP-LD5. The assessment of the antibody reactions against VP1 of CA16, CB3, PV1 and RV13 was conducted as above.

### Statistical analyses

Statistical analyses were conducted using the SPSS 17.0 and SAS 9.3 software. The statistical significance was tested using the Nemenyi non-parametric test or one-way ANOVA analysis. Differences between the measurements were considered significant at p-values less than 0.05.

## Electronic supplementary material

Supplementary Material

## References

[1] Tapparel C, Siegrist F, Petty TJ, Kaiser L (2013). Picornavirus and enterovirus diversity with associated human diseases. Infection, genetics and evolution: journal of molecular epidemiology and evolutionary genetics in infectious diseases.

[2] King, A. M. Q. *Virus taxonomy: classification and nomenclature of viruses: ninth report of the International Committee on Taxonomy of Viruses*. (Academic Press, 2012).

[3] Pallansch, M. A. & Roos, R., Enteroviruses: polioviruses, coxsackieviruses, echoviruses, and newer enteroviruses. In: Knipe, D. M., Howley, P. M. (Eds), Fields Virology, fifth ed. *Lippincott Williams & Wilkins, Philadelphia, pp*. **839–893** (2007).

[4] Ledford RM (2004). VP1 sequencing of all human rhinovirus serotypes: insights into genus phylogeny and susceptibility to antiviral capsid-binding compounds. Journal of virology.

[5] Arakawa M (2012). Molecular epidemiological study of human rhinovirus species A, B and C from patients with acute respiratory illnesses in Japan. Journal of medical microbiology.

[6] Palacios G, Oberste MS (2005). Enteroviruses as agents of emerging infectious diseases. Journal of neurovirology.

[7] Huang, X. *et al*. Epidemiological and Etiological Characteristics of Hand, Foot, and Mouth Disease in Henan, China, 2008–2013. *Scientific reports* **5** (2015). 10.1038/srep08904 PMC4354091 25754970

[8] McMinn PC (2002). An overview of the evolution of enterovirus 71 and its clinical and public health significance. FEMS microbiology reviews.

[9] Xing W (2014). Hand, foot, and mouth disease in China, 2008–12: an epidemiological study. The Lancet Infectious Diseases.

[10] Zhang T (2013). Epidemics and Frequent Recombination within Species in Outbreaks of Human Enterovirus B-Associated Hand, Foot and Mouth Disease in Shandong China in 2010 and 2011. PloS one.

[11] Tapparel C (2011). Rhinovirus genome variation during chronic upper and lower respiratory tract infections. PloS one.

[12] Wisdom A, Leitch EC, Gaunt E, Harvala H, Simmonds P (2009). Screening respiratory samples for detection of human rhinoviruses (HRVs) and enteroviruses: comprehensive VP4-VP2 typing reveals high incidence and genetic diversity of HRV species C. Journal of clinical microbiology.

[13] Henquell C (2012). Prospective genotyping of human rhinoviruses in children and adults during the winter of 2009–2010. Journal of clinical virology: the official publication of the Pan American Society for Clinical Virology.

[14] Watanabe A (2010). Rhinovirus species and their clinical presentation among different risk groups of non-hospitalized patients. Journal of medical virology.

[15] Ji H (2012). Seroepidemiology of human enterovirus71 and coxsackievirusA16 in Jiangsu province, China. Virology journal.

[16] Li W (2013). Seroepidemiology of human enterovirus71 and coxsackievirusA16 among children in Guangdong province, China. BMC infectious diseases.

[17] Luo ST (2009). Enterovirus 71 maternal antibodies in infants, Taiwan. Emerging infectious diseases.

[18] Tran CB (2011). The seroprevalence and seroincidence of enterovirus71 infection in infants and children in Ho Chi Minh City, Viet Nam. PloS one.

[19] Yu H (2011). Prevalence of antibodies against enterovirus 71 in children from Lu’an City in Central China. Japanese journal of infectious diseases.

[20] Zhu FC (2012). Retrospective study of the incidence of HFMD and seroepidemiology of antibodies against EV71 and CoxA16 in prenatal women and their infants. PloS one.

[21] Ding Y (2015). Characterization of the antibody response against EV71 capsid proteins in Chinese individuals by NEIBM-ELISA. Scientific reports.

[22] Ang LW (2011). The changing seroepidemiology of enterovirus 71 infection among children and adolescents in Singapore. BMC infectious diseases.

[23] Zeng M (2012). Seroepidemiology of Enterovirus 71 infection prior to the 2011 season in children in Shanghai. Journal of clinical virology: the official publication of the Pan American Society for Clinical Virology.

[24] Gao C (2016). Serological detection and analysis of anti-VP1 responses against various enteroviruses (EV) (EV-A, EV-B and EV-C) in Chinese individuals. Scientific reports.

[25] Yu WZ (2014). Poliomyelitis eradication in China: 1953–2012. The Journal of infectious diseases.

[26] Jiang SH (2008). Alternate Arrangement of PpL B3 Domain and SpA D Domain Creates Synergistic Double-Site Binding to VH3 and Vκ Regions of Fab. DNA and cell biology.

[27] Cao J (2011). Novel evolved immunoglobulin (Ig)-binding molecules enhance the detection of IgM against hepatitis C virus. PloS one.

[28] Zhang P (2013). A simple, universal, efficient PCR-based gene synthesis method: sequential OE-PCR gene synthesis. Gene.

[29] Liao W (2012). A designed Tat immunogen generates enhanced anti-Tat C-terminal antibodies. Vaccine.

[30] Hirota J, Shimoji Y, Shimizu S (2012). New sensitive competitive enzyme-linked immunosorbent assay using a monoclonal antibody against nonstructural protein 1 of West Nile virus NY99. Clinical and Vaccine Immunology.

[31] Mythili T, Rajendra L, Bhavesh T, Thiagarajan D, Srinivasan VA (2011). Development and Comparative Evaluation of a Competitive ELISA with Rose Bengal Test and a Commercial Indirect ELISA for Serological Diagnosis of Brucellosis. Indian journal of microbiology.

[32] Sharma N (2013). Detection of Francisella tularensis-specific antibodies in patients with tularemia by a novel competitive enzyme-linked immunosorbent assay. Clinical and Vaccine Immunology.

