# Supplementary material for: Non-neutralizing Antibody Responses against VP1 in Enterovirus A, B, C and Rhinovirus A species among Infants and Children in Shanghai
Source: Sci Rep. 2018 Apr 3;8:5455. doi: 10.1038/s41598-018-23683-x (PMC5882884; doi:10.1038/s41598-018-23683-x)
Supplement: Supplementary file 1 — Supplementary Material [file 41598_2018_23683_MOESM1_ESM.doc]

**Non-neutralizing Antibody Responses against VP1 in *Enterovirus A, B, C* and *Rhinovirus A species* among Infants and Children in Shanghai**

Yingying Ding^1#^, Bing Rui^1#^, Caixia Gao^1#^, Menghua Xu^2^, Lili Wang^1^, Chunyan Zhao^1^, Jie Bai^1^, Jinhong Wang^1^, Jin Xu^2*^, Wei Pan^1*^

^1^Department of Medical Microbiology and Parasitology, School of Basic Medicine, Second Military Medical University, No. 8 Panshan Road, Shanghai 200433, China.

^2^Department of Clinical Laboratory, Children's Hospital of Fudan University, 399 Wanyuan Road, Shanghai, 201102, China

^#^These authors contributed equally to this work.

*Send correspondence to Wei Pan, E-mail: pwpanwei@126.com; Jin Xu, Email: jin030101@aliyun.com

Table S1. Baseline characteristics of the study participants

|  | <28d | 1-6m | 7-12m | 13-24m | 25-36m | 37-48m | 49-60m | 61-72m |
| --- | --- | --- | --- | --- | --- | --- | --- | --- |
| **Number** | 27 | 80 | 54 | 62 | 47 | 33 | 38 | 23 |
| **Time** | 2015.5.5-2015.6.24 | | | | | | | |
| **Age** |  |  |  |  |  |  |  |  |
| Range | 1-28d | 1-6m | 7-12m | 13-24m | 25-36m | 37-48m | 49-60m | 61-72m |
| **Sex (%)** |  | | | | | | | |
| Male | 55.6% | 56.3% | 57.4% | 61.3% | 53.2% | 63.6% | 55.3% | 60.9% |
| Female | 44.4% | 43.7% | 42.6% | 38.7% | 46.8% | 36.4% | 44.7% | 39.1% |

Table S2. Primers for amplifying EV71 VP1, CA16 VP1, CB3 VP1, PV1 VP1 and RV13 VP1.

| Primers | Sequences (5’- 3’) | Description |
| --- | --- | --- |
| uEV71 | GCGCCGGGATCCG  GGTGACCGTGTTGCTGAC | The primer pairs uEV71/dEV71 were used to amplify EV71 VP1. uEV71 contains *Bam*H I restriction sites (underlined), dEV71 contains *Hin*d III restriction sites (underlined) |
| dEV71 | GCGCCGAAGCTT  CAGAGTAGTGATAGCAGT |
| uCA16 | GCCGCGCATATG GGTGACCCGATCGCTGAC | The primer pairs uCA16/dCA16 were used to amplify CA16 VP1. uCA16 contains *Nde* I restriction sites (underlined), dCA16 contains *Xho* I restriction sites (underlined) |
| dCA16 | GCCGGCCTCGAG CAGAGTAGTGATTTTGTC |
| uCB3 | GCGCGCGGGATCCG GGCCCAGTCGAGGATGCGGTAAC | The primer pairs uCB3/dCB3 were used to amplify CB3 VP1. uCB3 contains *Bam*H I restriction sites (underlined), dCB3 contains *Sal* I restriction sites (underlined) |
| dCB3 | GCGCCGGTCGAC AAAGGCACCAGTGTTTGTCA |
| uPV1 | CGGCCGCGGATCCG GGTCTGGGTCAGATGCTG | The primer pairs uPV1/dPV1 were used to amplify PV1 VP1. uPV1 contains *Bam*H I restriction sites (underlined), dPV1 contains *Sac* I restriction sites (underlined) |
| dPV1 | GCGCCGGAGCTCGA GTAGGTGGTCAGGTCTTT |
| uRV13 | CGGCCGCGGATCCG AACCCGGTTGAAAACTACGTTG | The primer pairs uRV13/dRV13 were used to amplify RV13 VP1. uRV13 contains *Bam*H I restriction sites (underlined), dRV13 contains *Sac* I restriction sites (underlined) |
| dRV13 | GGCCGCGAGCTCGA ACCGGTGGTGATGAATTT |
